# Unsung heroes in Ghana’s healthcare system: the case of community health volunteers and community health management committee

**DOI:** 10.1186/s12961-023-01099-y

**Published:** 2024-01-15

**Authors:** Samuel Egyakwa Ankomah, Adam Fusheini, Sarah Derrett

**Affiliations:** 1 Department of Obstetrics and Gynaecology, Komfo Anokye Teaching Hospital, P.O. Box 1934, Kumasi, Ghana; 2 Department of Preventive and Social Medicine, University of Otago, Dunedin, New Zealand; 3 Ngāi Tahu Māori Health Research Unit, Division of Health Sciences, University of Otago, Dunedin, New Zealand

**Keywords:** Community health management committee, Community health volunteers, Patient–public engagement, Community engagement, Health system improvement, Sub-Saharan Africa, Ghana

## Abstract

**Background:**

In Ghana, the community-based health planning and services (CHPS) policy highlights the significance of both community health management committees (CHMCs) and community health volunteers (CHVs) in the Ghanaian health system. However, research into their specific effects on health system improvement is scarce. Some research has focussed on the roles of the CHMCs/CHVs in implementing specific targeted health interventions but not on improving the overall health system. Therefore, this research aims to examine the role of the CHMCs and CHVs in improving the Ghanaian health system.

**Methods:**

The study was conducted in three districts in the Ashanti region of Ghana. A total of 35 participants, mainly health service users and health professionals, participated in the study. Data were collected using semi-structured individual in-depth interviews. Participants were selected according to their patient–public engagement or community health activity roles. Data were transcribed and analysed descriptively using NVIVO 12 Plus.

**Results:**

We found that the effectiveness of CHMCs and CHVs in health systems improvement depends largely on how members are selected. Additionally, working through CHMC and CHVs improves resource availability for community health services, and using them in frontline community health activities improves health outcomes.

**Conclusions:**

Overall, we recommend that, for countries with limited healthcare resources such as Ghana, leveraging the significant role of the CHMCs and CHVs is key in complementing government’s efforts to improve resource availability for healthcare services. Community health management committees and CHVs are key in providing basic support to communities with limited healthcare personnel. Thus, there is a need to strengthen their capacities to improve the overall health system.

## Background

Since the 1978 Alma-Ata Declaration on primary health care, there has been strong advocacy for community empowerment and involvement in designing, implementing and evaluating health activities [1, 2]. Empowering communities typically involves building their capacities and providing them with appropriate information to prioritize their healthcare needs and implement actions that seek to improve the healthcare system [3]. One way to empower communities is through community health management committees (CHMCs) and community health volunteers (CHVs) [4–6]. The CHMCs are voluntary, informal advisory groups, typically composed of 9–13 members and formed to create a clear link between the community and the formal healthcare system. They are to act in the interests of their community, especially in decision-making, and also supervise the activities of the CHVs [7]. CHVs are lay community health workers empowered to provide voluntary non-specialist basic healthcare support for their communities without receiving a regular salary or holding a ‘confirmed’ position within the formal health system [7, 8].

Past studies [4, 6, 8] have found that CHMCs and CHVs are pivotal in improving health outcomes. In Ghana, the Ghana Health Service and Teaching Hospitals’ Act, ACT, 525 of 1996 provides for health committees at various levels of the health system [9]. The introduction of the community-based health planning and services (CHPS) programme further provided greater recognition of CHMC and CHV roles in the Ghanaian health system [10]. The CHPS programme was introduced nationally in 1999 to increase healthcare access and empower local communities to have greater control over their healthcare activities [10]. It is a national primary health care (PHC) strategy aimed at mobilizing grassroots community resources and leadership to reduce health inequalities and remove geographical and physical barriers to healthcare access in Ghana, particularly for lower-income populations. At the centre of the CHPS implementation were the significant roles of the CHMCs and the CHVs [11]. Since implementation of the CHPS policy, it appears that no studies have specifically investigated the effect of CHMCs and CHVs on the Ghanaian healthcare system. Although some studies [12, 13] have investigated CHVs and CHMCs in Ghana, these have mainly focussed on the roles of the CHMC and CHVs in relation to particular targeted health programmes. For instance, one study [12] focussed on understanding the specific role of the CHMCs and CHVs in the distribution of azithromycin for the control of trachoma in Ghana; another [13] evaluated the impact of CHVs in the control of diarrhoea and fever among children. Whilst these studies provide useful findings about the roles of the CHMCs and CHVs within specific health programmes, there is a need to consider the role of the CHMCs and CHVs in improving the overall health system, particularly given the intent of the national health policies and legislation promoting patient–public engagement (PPE) activities for improved health in Ghana. This research, therefore, aims to examine the role of the CHVs and the CHMCs in improving the Ghanaian health system.

## Methods

### Study setting

The study was conducted in the Afigya-Kwabre South, Sekyere South and Asante-Akim North Districts in the Ashanti region of Ghana. The three districts have a combined population of about 440 000 distributed over a geographical area of approximately 1991 km^2^ [14–16]. Most residents in the districts are peasant farmers growing farm food crops such as maize, cassava, plantains, yams, citrus and vegetables on a subsistence basis [15]. The majority of the residents belong to the Ashanti ethnic group, and Twi is the most commonly spoken language in the districts. Regarding healthcare delivery, the Ashanti Regional Directorate of Health Services (RDHS) is administratively responsible for supervising healthcare services in the region. The RDHS also supervises the District Directorate of Health Services (DDHS) to implement health policy initiatives at the local level and supervises healthcare activities across all communities within the district [17, 18].

### Design and sampling

This study used a qualitative case study research design [19]. It was part of a broader qualitative study investigating the role of PPE in health system improvement in Ghana. Three districts with recognized good PPE structures, including CHMCs and CHVs, were identified. Overall, six communities (two from each district) were selected. In identifying potential districts and communities for this study, an informal survey via WhatsApp was conducted among health service administrators in the Ashanti region, Ghana, asking for recommendations of districts and communities with good PPE practices. The survey responses were complemented by suggestions from other key stakeholders in the Regional and District Health Directorate Offices whose roles were directly linked to supervising health service activities in the various districts/communities in the region. Participants were chosen using the maximum variation sampling to purposively select them from the national (macro/policy level), district and community health system levels. The national-level participants included representatives from the Ministry of Health. The district-level participants were District Directors of Health Services and Health Service Administrators. Various cadres of health professionals, such as midwives, community health nurses, CHPS coordinators and public health nurses from the district and the communities, were also selected. Participants were also selected from CHMC and CHV roles. The rest were community-based participants such as assemblymen/assemblywomen, traditional leaders (chiefs/queen mothers), and residents. The mix of varied participants was to provide additional perspectives, breadth and depth to the experiences and roles of the CHMCs and CHVs in health system improvement.

### Data collection

Prior to data collection, the chiefs and elders of each community were visited by the lead author to seek their consent for the study in accordance with local Ghanaian culture. The community-level participants involving the CHMCs, CHVs, assembly members, residents and traditional leaders were contacted through personal visits. The health professionals involving public health nurses, midwives, community health nurses, hospital administrators, district director of health services, and the representative from the Ministry of Health were also contacted through official letters and subsequently followed up with telephone calls. The lead author then conducted semi-structured interviews between December 2020 and August 2021. An interview guide developed specifically for this study was used to obtain the views, experiences and opinions of the participants on the role of CHMCs and CHVs in improving the health system. The interview guide broadly focussed on understanding how CHMCs and CHVs were selected and their specific roles in improving health and the overall health system. None of the selected participants withdrew from the study. Interviews lasted an average of 45–60 min. Before each semi-structured individual interview, the participant’s information sheet was read out to each participant. A local language translation of the participants’ information was also read to members who could not communicate fluently in English. Written consent was obtained from each participant before the commencement of the interview.

### Data analysis

The first author transcribed all 35 interviews. Those conducted in the Twi language were also directly transcribed into the English language by the first author with assistance from a native Twi language speaker. To maintain the anonymity of respondents, community sites are not named, all transcripts were de-identified and participants were assigned other unique identifiers and identified by their general roles [20]. Conduct of this study was approved by the Ashanti Regional Health Directorate of the Ghana Health Service following ethical approval from the University of Otago Ethics Review Committee, with clearance number 20/002.

Interviews were analysed and grouped into themes. Braun and Clarke’s six-phase guide on thematic analysis was employed to identify the themes [21, 22]. Transcripts were coded in NVivo Version 12 Plus [23]. In developing the key themes for analysis and interpretation, codes were first assigned to sub-themes and then grouped into major themes. The process also involved looking for consistent, co-occurring and overarching themes, including identifying different themes and sub-themes on the role of CHMCs and CHVs in improving the Ghanaian health system [24]. We duly documented the systematic process of collecting and analysing these research data and the processes and procedures involved in managing the data [20]. The second and third authors were involved in verifying the quality of data coding and reviewing interview transcripts to ensure data consistency and questioning the analysis process to assess for bias in relation to any preconceived ideas that may be influencing the analysis. A detailed research diary was kept noting all reflections, particularly after each interview or site visit.

## Results

Overall, across the six communities, 35 participants were interviewed. None of the invited participants declined to take part in the study. The participants included: traditional leaders (*n* = 3); people with dual CHMC–CHV roles (*n* = 11); residents/opinion leaders (*n* = 4); assemblymen/assemblywomen (*n* = 3); CHVs (*n* = 3); community health nurses/public health officers/midwives (*n* = 5); health administrators (*n* = 2); district directors of health services (*n* = 3); and representative from the MOH (*n* = 1) (Table 1).

**Table 1:** Participants’ role, case site (CS) and interview length

Participant	Afigya-Kwabre district		Sekyere-South district		Asante-Akim North district		Ministry of Health	Average interview length
	Semi-structured individual interviews							
Traditional leader (chief/queen mother)	1	1	1	0	0	0		45–60 min
Resident/opinion leader	1	0	0	1	1	1		45–60 min
Health professionals (e.g. community health nurses, public health officers, midwives)	1	0	1	1	1	1		45–60 min
CHMC & CHV (combined role)	2	1	2	2	2	2		45–60 min
Assemblyman/woman	0	2	1	0	0			45–60 min
CHVs only	0		1	0	1	1		35–60 min
Health facility manager/health administrator	0	1	0	0	1	0		60–85 min
District director of health services	1		1		1			45–60 min
Representative – Ministry of Health							1	45–60 min
Total	11		11		12		1	35

*CHMC* community health management committee, *CHV* community health volunteer

The gender distribution of participants was 70% male and 30% female. A greater number of the participants (46%) had tertiary education, 11% had secondary education, 40% had junior high school and 3% had no formal education. The average age of participants was 50 years (range 30–70 years). Of the 14 participants working as CHMC members and/or CHVs, 50% were aged 70 years or more. Except for one community, most CHMC members or CHVs were older than 50 years. The duration CHMC members or CHVs had served in their roles ranged from 2 years to 29 years.

The findings of the study were categorized into two sections. The first section is focussed on how CHMC and CHV members are selected. Nominations came from community durbars; community-based organizations, social and religious groups; district assembly elected members; traditional leadership recommendations; direct contact; and imposition by people in authority. The second part of the paper presents findings about the roles of CHMCs and CHVs in health system improvement. These include resource mobilization; accountability; support to healthcare workers; development of community health action plans; and supporting health education and other community health activities.

### Selection of CHMCs and CHVs

Despite the CHPS policy giving prominence to the role of CHMCs and CHVs in the Ghanaian health system, we found a lack of formal guidelines about how people should be selected for these roles. Unlike the regional and district health committees whose memberships were provided for in ACT 525 of 1996, the CHMCs/CHVs did not have such provisions. An interview with participants from the District Directorate of Health Services (DDHS), which also has the responsibility of supervising healthcare activities in the communities, indicated there was no official document regulating the formation and selection of members into the CHMC or CHVs. As a result, the communities mostly managed their own selection of members for CHMCs or CHVs. An interview with a district director of health service explained:

*In the absence of any document guiding their [CHMCs/CHVs] selection, we have created our own selection criteria for the communities in the selection of committee members or volunteers. We make sure that all important groups are represented. But of course, without any strict guide on these things, one is expected to have issues in terms of fully representing all interest groups in the community* (District Director of Health Service)

The approaches used to select members of the CHMC and the CHVs also seemed critical to their successful functioning. Thus, the absence of selection guidelines for the CHMCs and CHVs created some inconsistencies and challenges that affected their operations.

This study also identified six ways the communities used to select CHMH/CHVs as presented below.

#### Nominations at community durbars

Community durbars were regularly used for selecting CHMC members and CHVs. Durbars provided an opportunity for nominated CHMC members and CHVs to be confirmed and supported by the entire community and for screening the nominees. A participant shared their previous experience of selecting a CHV and CHMC member without the approval of the entire community:

*When we started the CHMC, I remember we selected someone who had questionable character… but he volunteered to help, so we selected him. However, we later saw that he could not function well both as a CHMC member and as a volunteer. Many people in the community deemed him unfit for the role because of his [problems]. If we had taken his nomination to a durbar, the community would have rejected him. Learning from some of these lessons, now every nomination goes to a community durbar for approval except mostly the traditional leader’s nominations* (CHMC member & CHV).

#### Nominations from community-based organizations, religious and social groups

Participants also noted that members of the CHMC and CHV were often drawn from community-based organizations (CBOs,) religious organizations and other social groups. Although no documented criteria on the composition of the CHMCs and the CHVs were found, nominations from these groups were key to the formation of CHMCs, which required a mix of community groups to be represented. Participants described nominations from Christians, Muslims, CBOs and other community social groups such as the market women, youth and the farmers as significant. These nominations, however, are not final, as some are contested by the public when subjected to a broader community approval during a community durbar. A CHV, who also serves as a CHMC member explained:

*When forming the CHMC, a lot goes into ensuring all the important religious and social groups are factored. We make sure everyone is represented. The prominent people [sic] are the churches and mosques. They are our most significant partners in this community health work, so we try to get their nominations to be part of this committee* (CHV & CHMC member).

#### District assembly elected members

Assembly members are elected representatives from the community or electoral area serving on the district assembly (local council). Each community mostly has one assembly member. We found that most assembly members were co-opted into the CHMC. Some participants noted that co-opting the assembly members into the CHMC was critical in ensuring they had a better appreciation of the health issues in the community:

*The assemblymen are automatically added to the CHMC, and they are replaced when new ones are elected. We just do that to ensure they understand the health challenges of the community and be able to also push that agenda at the District Assembly to improve our health delivery* (Resident/Opinion leader).

Whilst some participants were positive about this in their communities, others also mentioned that assembly members on the CHMC members had a less positive effect on PPE as they were generally passive members:

*Our assemblyman is supposed to be attending our CHMC meetings and even be part of the committee, but he does not seem to care about what goes on in this committee. I have not seen his impact on the CHMC. Maybe we should not include him as an automatic member. We should include assembly members who, in future, will be willing to be part of the committee and not just any assembly member who is elected* (CHMC member & CHV).

#### Nominations from traditional leadership

The traditional leadership was also found to be key in the formation of CHMCs and the selection of CHVs. Although there were differences in the practice among the six case sites, all CHMCs had at least one traditional council representative on the committee. The traditional representative was mostly a sub-chief who provided periodic feedback on the work of the CHMC and the CHVs to the traditional council. A CHMC member who represented the traditional council elaborated:

*On the committee, there is always a member of the traditional council team who represents our chief. The chief is required to be part of the committee, but because of his busy schedule, he mostly delegates one of us (the sub-chiefs) to represent him. At every traditional council meeting, he provides the chief with briefings on our activities, challenges and where he should personally intervene, he does* (CHMC member).

#### Direct contact

Many participants also mentioned that some CHMC members and CHVs were nominated through direct contact. We found that most community members who were considered suitable for the work of CHMC or CHV did not mostly volunteer to join. As a result, some were approached directly and persuaded to join. Participants explained that such persons were mostly people who already had the respect of the entire community and would be easily approved when their names were presented to the larger community. A CHV and member of the CHMC commented:

*There are many people on the committee we approached directly and appealed to them to join the CHMC or the CHV. Before we do this, as a committee, we ensure the person is someone who has an acceptable character and would be accepted by the entire community. Mostly, for such persons, if you don’t go to them, they may never come on board. Through this medium, we have been able to form a formidable CHMC and our CHVs are also made up of respectable personalities* (CHMC member).

In addition, apart from identifying and contacting such persons directly, others were also recommended by the existing members of the CHMC and CHVs.

*Again, people from our community make suggestions of those whom we can contact directly. Especially when we do not receive nominations from the churches, women’s groups and others, the individual community members sometimes suggest the name of people in these groups for us to contact. Often, we do that, and we are able to get a good representation of everyone for the committee* (CHMC member & CHV).

#### Imposition by people in authority

Many participants from across all six study sites noted how the establishment of the CHMCs was initially characterized by members nominated by people of high authority, particularly local politicians. This was due to some initial expectation of financial rewards associated with being a CHMC member or CHV. This type of nomination was identified as damaging the morale of many people who genuinely wanted to join the CHMC to improve the health system in the community:

*When we started the committee, it was bad. The whole selection process was influenced by people who thought there were some rewarding financial benefits from it. However, with time, all those people who were imposed on the committee, upon realizing there were no financial benefits, left the work. With time, we saw their true colours. They were just in for the money and not to voluntarily support the community. Out of the 13 members, only 5 of us who were inaugurated are still actively working. We had to bring others on board to make up the 13 people *(CHMC member & CHV).

Some participants also alluded to how the formation of CHMCs was influenced by some powerful individuals in authority whose sole aim was to benefit from the CHPS donor project funding to strengthen PPE activities in Ghanaian communities. A participant stated that these CHMCs existed in ‘name only’, and they collapsed soon after the funding ran out:

*We had this CHPS project funding to help community engagements and mobilizations. Because community engagement was mostly centred around the CHMCs, most funding activities supported the CHMCs and CHVs. I know that many CHMCs were formed just to enjoy that money. Immediately after the funding ended, those CHMCs ceased to exist. Some of our big men, both politician and our own health staff, planted community members into CHMCs without any work but just to benefit from the funding* (District Director of Health Services).

### Roles of CHMCs and CHVs in health system improvement

We noted from this study that most engagement activities, either at the regional, district or community levels, were supposed to be implemented through health committees or volunteers. However, as indicated earlier, these committees were found to function mainly at the community level, despite statutory provisions for effective patient–public engagement (PPE) across all health system levels in Ghana. For instance, at the regional and district health system levels, the Regional Health Management Committee and the District Health Management Committee are expected, by law, to function adequately to improve the health system. However, results from our study revealed the CHMCs were the only health committee that were functional. A district director of health services explained at an interview:

*It would be good to have the public involved in major health policy decisions right from the Ministry and even the highest decision-making body of Ghana’s Health Service. But I do not think we are there yet* (District Director of Health Services).

A representative from the Ministry of Health also commented:

*You see, it will be good to have effective user involvement even in our plans and policy developments right from the Ministry to the community, but that has not happened yet. We see PPE as more of a community activity than a national one. So, for now, such user engagement strategies have been implemented only in the communities* (Representative, MOH).

We found the CHMCs and CHVs played important roles in improving the health system across five key themes: (1) resource mobilization; (2) accountability; (3) support to health workers; (4) develop community health action plan (CHAP); and (5) supporting health education and other health activities.

#### Resource mobilization

Mobilizing resources for community health activities was mentioned by many participants as a core function of the CHMCs. Although making resources available for community health activities is primarily the duty of the government, CHMCs had taken this up to complement the government’s efforts. For instance, in one of the communities, a CHMC member who was also a traditional leader donated a piece of land for the construction of the community clinic. In addition, members of that CHMC manually dug the ground to lay pipes for treated water to reach the community’s clinic. Members of the CHMC also provided 24-h security for the community clinic. A CHMC member commented as follows:

*We have done so many things as a committee to ensure the clinic has adequate resources for their work. From the day we conceived the idea to have a clinic in the community, one of our members, who is also a sub-chief here, donated his land for the clinic. Not only that but we, as CHMC, also volunteered to work as construction workers. Even after the construction, when the government could not bring water to the clinic, we used a pickaxe to dig the hard ground to connect water to the clinic. Currently, I and another CHMC member still volunteer as security personnel for the clinic. This is how committed our committee is to support our community to improve our health* (CHMC member & CHV).

In another site, community representatives on the hospital board were instrumental in lobbying renowned community members to donate an X-ray and other laboratory machines to the hospital. An interview with the Health Administrator of the facility highlighted additional support they had received from the community representatives and their key roles in mobilizing resources for the hospital:

*Aside from what I mentioned earlier, the traditional leader on our hospital board again spoke to a renowned dental surgeon who also hails from this community but resides in Europe. After that conversation, I was invited to Europe to present our case before a donor organization. Following that, we took delivery of several dental chairs and other dental equipment. Now, starting from October 2022, we are commencing the training of community oral health officers in this facility. This alone should tell you how these representatives have been supportive to us* (Health Administrator).

Similar examples were found in other communities where the CHMC led the communities to mobilize funds to renovate existing buildings into new community clinics. In addition, the CHMC members further organized durbars and embarked on door-to-door campaigns to mobilize funds from the community to purchase medicines for the clinic. This was again given a further boost by another CHMC member who appealed to pharmaceutical companies to support the community’s clinic with various medicines for their clinic operations:

*One of the CHMC members who had worked in a pharmaceutical company contacted some of these companies and provided us with the rest of the medicines we needed. Unlike other places, we do not send patients outside the facility to go and buy drugs. We have them here unless your condition is beyond us* (Midwife).

Similar examples were found across all the case sites. A district director of health services in an interview summarized the importance of CHMCs, particularly in mobilizing resources to support community health activities:

*You know, in our part of the world, we wait for the government to do everything for us. Unfortunately, the government cannot do it all. There are many children who are calling for the same food, and the resources to satisfy these needs are scarce. That is the more reason why we need to take the CHMCs seriously. They have done amazingly well in many communities. Where the government has not been able to provide the needed resources, they find innovative means to mobilize resources for that. This has really improved healthcare in the communities* (District Director of Health Service, 1).

#### Accountability

Accountability was one of the major roles of both the CHMCs and the CHVs. Among the key things mentioned was the implementation of a scorecard system, which empowered the CHMCs to provide quarterly feedback to the DDHS about services rendered to the community. Scorecards, although not implemented across all case sites, were found helpful in communities using them:

*If you look at the scorecard system, I see it as a good way of forcing accountability from the health workers. Periodically, we invite the health workers to our CHMC meetings to present to us the state of healthcare in the community, and we also ask critical questions on behalf of the community. For the fact that they know we provide quarterly feedback to the district health office, they normally do not miss our invitations* (CHMC member & CHV).

Again, in some case sites, CHMC and CHVs had a special day each week on which they met patients and the larger community for feedback on the quality of care received from the clinic, including the health workers’ attitude towards them. The CHMCs and the CHVs discussed this feedback with the health workers, and measures were instituted to improve the gaps in service delivery.

*Every Thursday at the clinic, we call it the volunteer day, where the community reports all issues, complaints, suggestions and any other form of concerns about healthcare to the members of the CHMC or CHVs* (Resident/Opinion leader).

However, despite these key accountability roles, many participants, especially the CHMC members and the CHVs, expressed much disappointment in the reluctance or refusal of the health workers to be financially accountable to the community representatives. Participants noted that the health workers had continuously resisted any form of financial accountability, despite the community being a major stakeholder in resourcing the operations of the clinic:

*We have been supporting this clinic all this while. As a matter of fact, this building was a [defunct company], and we lobbied for it and converted it into a clinic. Following that, we had to mobilize money in renovating it and the entire community was involved in offering communal labour to support this. So, I am not afraid to say it is our clinic. However, we want to know the financial situation of the clinic. It helps us to understand how the little revenues they make there are utilized. As it is now, we are kept in the dark on these issues, and I must say it is not the best. Maybe you can talk to them about it. We are not coming to take the money, but we need to know how the little they generate there is spent. It helps us to appreciate how we can financially support them, but the health workers will not agree to let us know* (Queen Mother, CS 3).

#### Support to healthcare workers

The CHMCs are crucial in providing various forms of support to health workers. For instance, we found that CHMCs supported newly posted health workers in their communities with accommodation and food supplies, which was key for the initial integration and settlement into the community. As a result of this support, there was improved health worker retention in these communities which were considered remote:

*The CHMC mostly ensures all new healthcare personnel posted to this community have decent accommodation and even ensure they are comfortable in their entire stay in the community. So, in our community, before health workers are posted, the district directorate usually discusses with the chairman of the CHMC so that we try to prepare for the person’s coming. Mostly, when the person arrives, a delegation of CHMC members first meets the health worker, and then we send him/her to the accommodation. The next day, we introduce him/her to the chief’s palace before being introduced to the entire CHMCs officially. My duty as a member of the CHMC is to do everything possible to ensure they (health workers) enjoy their stay here* (CHMC member & CHV).

While newly posted health workers may experience difficulty integrating into the new environment’s culture, the CHMC was reported to be significant in easing their stay. They helped the health workers understand the community’s way of life and the best way to live in peace and harmony. A participant explained:

*The CHMC supports the health staff to make their work in the community easy. We, as community members, understand our issues better. They are also trained health workers, so they are good at what they do. But when it comes to our culture, behaviours, or ways of living as a people, they don’t know. So, we are here to support them to deliver effective healthcare to us. That healthcare must be done in a way that does not offend our thinking, ways of life, etc. They need to also understand us to be able to do their work effectively. So, this is our biggest role here, and so far, we have delivered that well* (CHMC member & CHV).

In addition, many participants acknowledged that the CHMC and CHVs helped resolve conflicts between health workers and community members. The participants noted that the CHMCs and CHVs, through their roles, work closely with both the general community and healthcare workers. Therefore, this enables them to play an intermediary role in resolving most conflicts and misunderstandings between community members and health workers:

*I remember a time when the whole community was outraged and came down to attack a midwife in the clinic because they believed she had contributed to the death of a neonate. Unfortunately, the midwife was also equally angry because she felt the patients and relatives had insulted her unjustifiably despite all her efforts to save the mother and baby. This almost got the midwife demoralized to leave our community. Our committee and some of our volunteers came in and investigated the case. We later found a better solution to the problem. There have been a few other cases like that in which the committee or the volunteers had to intervene to bring peace between our health staff and some of our community members* (CHMC member & CHV).

#### Development of community health action plans (CHAP)

The CHMCs were involved in the development of Community health action plans (CHAP), which offered communities an opportunity to be part of planning healthcare services and decisions. Participants opined that CHAP mostly provided annual direction for community health activities:

*The CHAP is one important thing the CHMCs do. CHAP contains the annual health plans of the community. It also contains the health needs to be addressed that year, the resources needed to tackle these, and the means of implementation and verifications. As part of the implementation, there are timelines that go with every action and how all of these would be evaluated at the end of the year with the CHMC. The CHMCs are supposed to do this, although with the assistance of staff from our office* (District Director of Health Service 1).

Despite the development of CHAP being considered a major role for the CHMCs, only a few communities had CHAP in place. Some participants were of the view that health workers mostly led the development of CHAP primarily from the DDHS offices instead of community representatives. As a result, the final plan did not consider most community inputs into this health project. A participant explained:

*At our very first CHMC training meeting at the district health office, we were told that one of our duties was to develop an action plan for the community. As a secretary of the committee, I took it up. We were specifically trained on how to prepare the action plan. However, anytime we develop our plan, the district health people will take most of the information out. They will rather put in their own information* (CHMC member).

#### Support health education and other community health activities

Participants also reported various activities of the CHVs that contributed to supporting health education and community health. These include assisting community health nurses or public health nurses with home visits, outreach services and general health education. In addition, they also followed up on all non-attendees for their medical appointments:

*Another activity undertaken by CHVs in the communities is that they do community outreach, sensitization and education, visit individual homes of patients to administer vaccines and immunizations and offer other forms of home care services to patients with chronic diseases. Of course, they do these with us, but in places where there are no community health nurses, they do these alone, too. Also, due to the stigma of HIV/AIDS, they help deliver medicines to some of our HIV/AIDS patients* (Community Health Nurse).

Additionally, a few participants indicated that the CHVs supported disease control officers in carrying out community disease surveillance as well as reporting on diseases and other health issues in the community or a particular locality:

*They (the CHVs) also support the disease control staff in the communities to conduct disease surveillance and report on them. In instances where we don’t even have disease control staff, most of the CHVs have been trained to do this for us* (District Director of Health Services).

Another important role of the CHVs was assisting communities in compiling and regularly updating community health registers and profiles. The register and profile made it easy to compile reports on the health status of the communities as well as implementing community-based health programmes:

*The volunteers and some of the committee members also help to compile the community-based registers which contain the detailed health profile of the community, and it helps us in our periodic health reporting. Most of our decisions and national reports are based on the information contained in the report the volunteers compile for us* (Public Health Nurse).

Lastly, with many village communities located in the hard-to-reach areas of the districts, CHVs were mostly trained to support in providing some first aid treatments for minor diseases and injuries and quickly refer to the appropriate community health nurse or clinic.

*Already, we have low numbers of community health nurses in most of the villages. So, in these deep villages, we have trained the CHVs to deliver basic first aid treatments for minor injuries and some diseases and quickly refer these patients to the nearest community health nurse* (District Director of Health Service).

## Discussion

This study provides a detailed understanding of the significant role of CHMCs and CHVs in improving health and the overall health system in Ghana. Whilst we note that CHMCs and CHVs play an important role in improving the health system, their selection process is also crucial for effective functioning. We found that the processes for selecting CHMC members and CHVs were not provided in the CHPS policy, which formally introduced these roles to the Ghanaian healthcare system. The findings of this study, however, have highlighted a range of strategies that were key for the selection of CHMC members and CHVs. Firstly, we noted that community durbars were an important strategy for selecting (or objecting to) community health representatives. This study established that community health representatives selected through durbars had a wider community support. This finding was consistent with earlier studies [25–27] conducted in other Sub-Saharan African countries, which similarly argued that Community Advisory Board members selected through durbars had greater community support than those selected using different approaches. However, a study [28, 29]conducted in Nepal in which durbars were found as a key social accountability tool did not offer community members the opportunity to select or nominate their health committee members.

Nominations of CHMC/CHVs from various CBOs, religious and other social groups were also considered significant, as they improved the right mix of representation on the committee and ensured a range of community groups were represented. Significantly, this helped provide equal opportunity for minority groups to be a part of decision-making to improve the health system. In a scoping review [8] of PPE strategies in Sub-Saharan Africa, it was found that nominations from the CBOs and religious groups were key to improving the quality of membership in the CHMCs and the CHVs. However, we found that such nominations also required a larger community acceptance in a durbar.

Other strategies employed in selecting members for the CHMC and CHV included nominations from the traditional leaders, co-opting elected members of the district assembly and directly approaching key individuals. Nomination from traditional leaders has been widely reported in the literature [11, 30] as an important way to integrate into the CHMC. This study noted that incorporating traditional leadership in the CHMC increases its acceptability in the community and improves its ability to influence decision-making.

Regarding the roles of the CHMCs and the CHVs, we found that one of their recognized roles included mobilizing resources to support community healthcare activities. This study found that many communities have, through the leading efforts of the CHMCs and the CHVs, mobilized resources to construct new community health facilities and acquired new medical equipment to support healthcare delivery without government support. These efforts have significantly improved health and the healthcare system and it is particularly crucial for a resource-poor country such as Ghana, which spends more than 80% of its healthcare budget allocation on personnel emoluments and less than 20% on equipment and infrastructure [31]. As a result, most district health directorates are not adequately resourced to support healthcare activities in their sub-districts and communities [32]. Therefore, the roles played by the voluntary CHMCs and the CHVs in mobilizing resources to support healthcare activities in the communities are considered significant. Although other studies [28, 33, 34] found that many countries restricted the roles of these health committees to resource mobilization, we found that CHMCs and CHVs in Ghana contributed in other ways towards improving the health system.

In addition to resource mobilization, the CHVs and some CHMC members across all case sites were found to have also supported the health workers in delivering direct healthcare services to their community. With an unequal distribution of healthcare personnel in Ghana, particularly between rural and urban areas, most rural Ghanaian communities need adequate health workers, especially community health nurses [31, 35]. Therefore, the supportive role of the CHVs, which could be likened to China’s barefoot doctor scheme[36], was significant in complementing the shortage of health workers in rural areas. This study found that in the difficult and hard-to-reach communities where there were few (or no) health workers, the CHVs were trained to support the delivery of healthcare services adequately.

The CHMCs also provided other forms of support and incentives for health workers posted to their communities. This support was mainly in the form of accommodation and food supplies. For example, in four communities, it was found that clinical nursing and medical students posted for community internships were mostly hosted by the communities through the CHMCs and the CHVs. This was reported to make the communities attractive to newly qualified health workers and contributed to their high retention. Studies conducted in Malawi and Tanzania also had similar findings [33, 37]. However, this support was not reported to have extended to providing accommodation or supporting health workers with foodstuffs, as found in our study. Significantly, this community-level support improved rural health worker retention in the communities, complementing the Government of Ghana’s effort to improve rural health worker retention in the country [38].

Lastly, the CHMCs and the CHVs played crucial roles in resolving conflicts and misunderstandings between community members and health workers. For example, as observed from two communities, there were key incidences that resulted in the communities feeling dissatisfied due to health workers disrespecting their traditional systems of providing healthcare. Consequently, the communities boycotted the services of the clinic. As noted by Halian et al., occasional reports of conflict between health workers and community members are not unusual [39]. However, the ability to resolve these conflicts promptly and effectively is significant. Therefore, as noted in this study, the significant and timely role of the CHMCs and the CHVs in resolving these conflicts effectively sustained and improved health worker–community relationships. Other studies conducted in Ghana and Malawi also reached similar conclusions [11, 40].

## Conclusions

Overall, the findings of this study have highlighted the critical roles played by CHMCs and CHVs in improving the Ghanaian health system. While we recognize the significant role played by these community health representatives in health system improvement, we also note that their effectiveness can hinge on how members are selected. Thus, we recommend a workable guideline for the selection of CHMC members and CHVs, particularly taking into consideration having the required mix of community groups being represented.

Again, despite legislation and policies existing for effective functioning of health committees across all levels of the Ghanaian health system, implementation has so far occurred at only the community levels. This seems to have limited the overall effect of the health committees to only few communities with well-functioning CHMCs or CHVs. We therefore recommend enforcing the existing legislation and policies that allow for lay involvement in decision-making across all levels of the health system to improve the wider health system.

Finally, the findings of this study have shown that working through the CHMCs and CHVs is critical to improving resource availability for community health services, especially in resource-constrained countries such as Ghana. We found that using the CHMC and CHVs in frontline community health activities also improved health worker retention and overall health outcomes. Thus, we suggest there is a need to actively strengthen PPE activities across all levels of the health system to deliver an improved health system.

## Data Availability

The data supporting the findings of this study are available from the corresponding author upon reasonable request.
